# Association between neighborhood deprivation and type 2 diabetes risk among ADHD patients: a nationwide population-based cohort study

**DOI:** 10.3389/fpubh.2025.1609551

**Published:** 2025-09-11

**Authors:** Yiman Li, Huifang Yang, Kristina Sundquist, Jan Sundquist, Yuhong Zhang, Xinjun Li

**Affiliations:** ^1^School of Public Health, Ningxia Medical University, Yinchuan, China; ^2^Key Laboratory of Environmental Factors and Chronic Disease Control, Ningxia Medical University, Yinchuan, China; ^3^Center for Primary Health Care Research, Department of Clinical Sciences, Lund University, Malmö, Sweden; ^4^University Clinic Primary Care, Skåne University Hospital, Malmö, Sweden; ^5^Departments of Family and Community Medicine and of Epidemiology, The University of Texas Health Science Center, Houston, TX, United States; ^6^Center for Community-Based Healthcare Research and Education (CoHRE), Department of Functional Pathology, School of Medicine, Shimane University, Matsue, Japan

**Keywords:** type 2 diabetes, attention-deficit/hyperactivity disorder, neighborhood, population-based, Sweden

## Abstract

**Objective:**

Both attention-deficit/hyperactivity disorder (ADHD) and neighborhood deprivation have been previously associated with an increased risk of type 2 diabetes (T2D). However, the potential association between neighborhood deprivation and T2D in ADHD patients remains underexplored. Our aim was to study the potential effect of neighborhood deprivation on incident T2D in patients with ADHD.

**Methods:**

This study included adults (*n* = 246,515) with ADHD who were followed in Sweden from 2001 to 2018 for incident T2D. The relationship between neighborhood deprivation and incident T2D was examined using Cox regression analysis, reporting hazard ratios (HRs) with 95% confidence intervals (CIs). All models were stratified by sex and adjusted for age, educational level, family income, employment status, region of residence, immigrant status, marital status, family history of T2D, and comorbidities. Patients with ADHD residing in neighborhoods with high or moderate deprivation were compared to those in neighborhoods with low deprivation (reference group).

**Results:**

A significant association was observed between neighborhood deprivation and T2D in patients with ADHD. Among patients with ADHD residing in highly deprived neighborhoods, the HRs were 1.37 (95% CI: 1.22–1.53) for men and 1.84 (95% CI: 1.61–2.12) for women, compared to those in low-deprivation neighborhoods. After adjusting for potential confounders, the association remained significant, with HRs of 1.19 (95% CI: 1.06–1.34) in men and 1.48 (95% CI: 1.28–1.70) in women residing in highly deprived neighborhoods.

**Conclusion:**

The increased incidence of T2D among patients with ADHD residing in deprived neighborhoods raises significant clinical and public health concerns. These findings could assist policymakers in allocating resources within primary healthcare settings and provide guidance for clinicians working with patients in deprived neighborhoods.

## Highlights

**What has this study found?**

- Graded relationship in higher deprivation with higher T2DM risk among ADHD patients.
- Deprived areas may be an independent risk factor for T2DM among ADHD patients for both men and women

**What are the implications of the study?**

- These findings could assist policymakers in allocating resources within primary healthcare settings and provide guidance for clinicians working with patients in deprived neighborhoods.

## Introduction

Attention-deficit/hyperactivity disorder (ADHD) is a neurodevelopmental condition that affects 2 to 7% of individuals globally (1). Recent studies suggest that ADHD may independently elevate the risk of developing type 2 diabetes (T2D) (2, 3). A longitudinal study conducted in Taiwan analyzed data from the National Health Insurance Research Database, including over 35,000 individuals with ADHD and more than 70,000 matched controls. The results showed that young adults and adolescents with ADHD were nearly three times more likely to develop T2D compared to those without ADHD (4). Another cohort study conducted in Sweden assessed data from more than 5.5 million adults aged 18 to 64 years. It concluded that adults with ADHD were 70% more likely to develop T2D than those without the disorder (5).

Several factors may explain this increased risk, such as lifestyle factors, medication side effects, and overlapping genetic or environmental influences (6). Socioeconomic status and neighborhood deprivation have an impact on both ADHD and T2D. For example, studies have shown that the incidence of ADHD is higher in deprived neighborhoods than in more affluent areas, even after adjusting for individual characteristics (7–9). Additionally, neighborhood deprivation has been associated with higher rates of T2D incidence (10), as well as an increased prevalence of key risk factors, including obesity (11). Furthermore, individuals residing in deprived neighborhoods may often face fewer health-promoting resources in many countries worldwide, and challenges in accessing primary care (12, 13). Considering all of this, it is plausible that the neighborhood effect on T2D (10, 14) may influence the burden of T2D in individuals with ADHD.

Both ADHD and neighborhood deprivation have been previously associated with an increased risk of T2D (4, 15). However, the potential association between neighborhood deprivation and T2D with ADHD remains underexplored. A deeper understanding of this association is required, as uncovering a meaningful relationship could enable more targeted strategies to identify individuals with ADHD who are at particularly high risk for T2D. Identifying a clear link could lead to the development of targeted interventions which could help recognize individuals with ADHD who are at increased risk for T2D due to socioeconomic or environmental disadvantages.

It is important to keep in mind that, unlike many countries such as the US, neighborhood deprivation in Sweden occurs within a context of strong social policies, including universal healthcare coverage and urban planning. Notably, previous Swedish research has found that while health-promoting services (e.g., healthcare resources, cultural resources, and sports facilities) are more prevalent in deprived areas (12), these neighborhoods also feature a higher density of health-damaging exposures, such as fast-food outlets, liquor stores and bars (16). This duality contrasts with patterns observed in the US and other countries, where deprived neighborhoods often face lower access to healthcare resources and other health-promoting services in addition to the higher density of health-damaging exposures.

This study aims to assess the impact of neighborhood deprivation on T2D risk in ADHD patients, specifically examining whether there is a difference in T2D incidence between individuals with ADHD living in deprived neighborhoods versus those residing in more affluent neighborhoods. By addressing this question, the study aims to uncover new insights into how social factors influence chronic physical and psychiatric conditions, while adjusting for individual characteristics such as age, education, income, and comorbidities. National registers and primary healthcare data were integrated to explore these associations. This combined approach has not previously been employed to explore potential risk factors for T2D in patients with ADHD.

## Methods and materials

### Design and setting

This nationwide cohort study examined the association between neighborhood deprivation and the risk of developing T2D in adults diagnosed with ADHD. The study included individuals diagnosed with ADHD between 2001 and 2018. Baseline was defined at the point of ADHD diagnosis, and the analysis compared the impact of different levels of neighborhood deprivation (low, moderate, and high), with individuals living in more affluent neighborhoods serving as the control group.

Data were sourced from Sweden’s national registers, which provide extensive individual-level information on all residents. The study adhered to the STROBE guidelines for cohort studies to ensure a robust and transparent methodological approach. Conducted by researchers at Lund University, this work sheds light on the role of social and environmental factors in shaping health outcomes for individuals with ADHD, particularly regarding their risk of metabolic conditions like T2D.

### Study population

Using data from Sweden’s National Patient Register (17) and primary healthcare records (18), researchers identified all individuals diagnosed with ADHD between 2001 and 2018. Diagnoses were based on ICD-10 codes (F90). From this group, a total of 250,386 patients were identified. Exclusions were made for 516 individuals (0.2%) who had a prior diagnosis of any types of diabetes recorded between 1998 and 2000 (under ICD-10 code E10–E14), as well as 3,170 individuals (1.3%) who developed T2D before their ADHD diagnosis during the study period. After these exclusions, 246,515 patients (representing 98.5% of the initial cohort) were included in the final study population (Supplementary Figure S1). ADHD medications were identified using Anatomical Therapeutic Chemical codes in the Swedish Pharmacy Register and included amphetamine (N06BA01), dexamphetamine (N06BA02), methylphenidate (N06BA04), and lisdexamfetamine (N06BA12). The Swedish Pharmacy Register started on July 1, 2005, and includes all medications prescribed and dispensed nationwide.

### Data source

This study utilized comprehensive nationwide registers (17) and primary healthcare data (18) from Sweden, including individual-level details for the entire population. The data encompassed age, sex, socioeconomic status, geographic region of residence, healthcare diagnoses, family relations, hospital admission dates, emigration details, and causes of death. Medical conditions were identified using primary healthcare data (1990–2018) from 20 of Sweden’s 21 healthcare regions and the National Patient Register, which supplied outpatient (2001–2018) and inpatient (1964–2018) records maintained by the National Board of Health and Welfare (Socialstyrelsen). Additional data were sourced from the Cause of Death Register (19) (1961–2018) and the Total Population Register (20) (1968–2018), both of which are nearly complete for Sweden’s population. Linkages across these databases were facilitated using Sweden’s unique civic registration number, assigned to all residents at birth or immigration, and replaced with pseudonymized serial identifiers to ensure privacy.

### Outcome variables

This study utilized data from the National Patient Register to identify T2D diagnoses. For the purpose of the research, a first-time hospital admission with T2D diagnosis, classified under ICD-10 code E11 during the study period, was considered an incident case. T2D medications were identified using Anatomical Therapeutic Chemical codes in the Swedish Pharmacy Register and cods A10.

### Neighborhood level variable

Neighborhood deprivation was the primary exposure and was assessed at baseline. The assessment of this variable was feasible because all adults living in Sweden have been geocoded to small geographic administrative units with boundaries defined by homogeneous types of buildings. These neighborhood units, referred to as small area market statistics (SAMS) (21), have an average population of 1,000 to 2,000 people and were used as proxies for neighborhoods. The Neighborhood Deprivation Index (NDI) was calculated as a summary measure to characterize neighborhood-level deprivation. Deprivation indicators used in previous studies to describe neighborhood environments were identified, and a principal components analysis was employed to select the relevant indicators from the Swedish national database. The following four variables were selected for individuals aged 25–64: (1) low educational attainment (<10 years of formal education); (2) low income (income from all sources, including interest and dividends, defined as less than 50% of individual median income); (3) unemployment (not employed, excluding full-time students, those completing compulsory military service, and early retirees); and (4) social welfare dependency. The calculation of the neighborhood deprivation index was based on the population aged 25 to 64 years since this age group (i.e., the working population) was considered to be more socioeconomically active than other age groups. All four deprivation variables loaded onto the first principal component with similar loadings (+0.47 to +0.53) and explained 52% of the variation among these variables. A *Z*-score was calculated for each SAMS neighborhood. The *Z*-scores were weighted by the coefficients for the eigenvectors and then summed to create the index. The index was categorized into three groups: below one standard deviation (SD) from the mean (low deprivation), above one SD from the mean (high deprivation), and within one SD of the mean (moderate deprivation). Higher scores reflect more deprived neighborhoods. The data needed for the neighborhood deprivation variable was collected from the Total Population Register at the time of ADHD diagnosis, a total of 6,183 neighborhoods were included (Supplementary Table S1).

### Individual level variables

All individual-level variables were assessed at the time of an ADHD diagnosis and included in the analysis, as they may function as confounders in the relationship between neighborhood deprivation and T2D in patients with ADHD due to their association with both the predictor and the outcome*. Comorbidities* were identified from the National Patient Register during the study period and were defined as follows: obesity (E65–E68); depression (F32 and F33); and anxiety (F40–F43). Data on individual-level sociodemographic factors were collected from the Total Population Register. *Age* was treated as a continuous variable for individuals. *Educational attainment in parents* was categorized into three groups based on the highest level completed: completion of compulsory school or less (<9 years), practical high school or some theoretical high school (10–11 years), or theoretical high school and/or college (≥12 years).

*Family income* was calculated as the sum of all family members’ incomes, multiplied by the individual family member’s consumption weight (i.e., where small children were given lower weights than adolescents and adults), and divided by the total consumption weight of the family members. *Country of origin* was categorized as “born in Sweden” or “born outside Sweden.” *Marital status in parents* was defined as “never married, widowed, or divorced” or “married/cohabiting.” *Region of residence* was classified as “small towns/rural areas,” “middle-sized towns,” or “large cities” (Stockholm, Gothenburg, and Malmö).

*Family history of type 2 diabetes* was defined as first-degree relatives (father, mother and siblings) with and without a diagnosis of type 2 diabetes during the study period.

### Statistical analysis

Descriptive characteristics were computed for the study population and its variables. Person-years were calculated from baseline, defined as the point at which individuals were diagnosed with ADHD during the study period, until the first diagnosis of T2D, death, emigration (using data from the Total Population Register), or the study period’s conclusion on 31 December 2018.

Cox proportional hazards models were employed to analyze associations between neighborhood deprivation, covariates, and the time to the first diagnosis of T2D. The stratified model provided hazard ratios (HR) and 95% confidence intervals (95% CI) for T2D, adjusted for individual-level variables. Analyses included three models: Model 1 adjusted for age; Model 2 for age and individual-level sociodemographic factors; and Model 3, which incorporated all covariates. Analyses were conducted separately for men and women. The proportional hazard assumptions were also checked by plotting the incidence rates over time and calculating Schoenfeld (partial) residuals; no meaningful departures from these assumptions were identified. Interaction tests were performed to examine whether the association between neighborhood deprivation and T2D among patients with ADHD was affected by individual-level variables. A sensitivity analysis was performed, which included patients with antidiabetic treatments as a proxy for T2D, defined as ATC-codes (A10) retrieved from the Swedish Prescription Register from July 1, 2005, and December 31, 2018. All individuals that were prescribed and picked up an insulin or an oral antidiabetic agent during the entire time period were included in this sensitivity analysis. An additional sensitivity analysis was performed for ADHD patients identified in medication treatments and hospital diagnosis, separately. Another sensitivity analysis was conducted after excluding individuals with residential mobility during the study period. Finally, an additional analysis was conducted for the association between neighborhood deprivation and T2D by the number of ADHD diagnoses. All statistical analyses were conducted using SAS 9.4 (SAS Institute Inc.; Cary, NC, United States).

## Results

Table 1 shows the study population which includes a total of 246,515 patients with ADHD. Over the follow-up period (mean follow-up = 5.4 years), 2,622 cases of T2D were reported in men and 1,953 in women. A gradient was evident, with higher cumulative incidence rates of T2D observed as neighborhood deprivation increased, a trend that also appeared across most age groups.

**Table 1:** Distribution of population, number of cases, and cumulative rates (per 100) of type 2 diabetes of ADHD patients, 2001–2018.

	Population		Type 2 diabetes cases		Cumulative rates (%) of diabetes by neighborhood deprivation		
	No.	%	No.	%	Low (*n* = 63,000)	Moderate (*n* = 129,354)	High (*n* = 54,161)
Total population	246,515		4,575		1.3	1.9	2.3
Gender							
Males	149,242	60.5	2,622	57.3	1.4	1.8	2.2
Females	97,273	39.5	1,953	42.7	1.3	2.1	2.6
Age (years)							
<20	139,949	56.8	1,293	28.3	0.8	1.0	1.0
20–29	43,576	17.7	651	14.2	1.1	1.5	1.8
30–39	29,976	12.2	829	18.1	1.7	2.8	3.7
40–49	20,674	8.4	917	20.0	2.9	4.5	5.9
50–59	9,118	3.7	592	12.9	4.9	6.7	7.8
≥60	3,222	1.3	293	6.4	8.0	9.6	9.1

**Figure 1:**
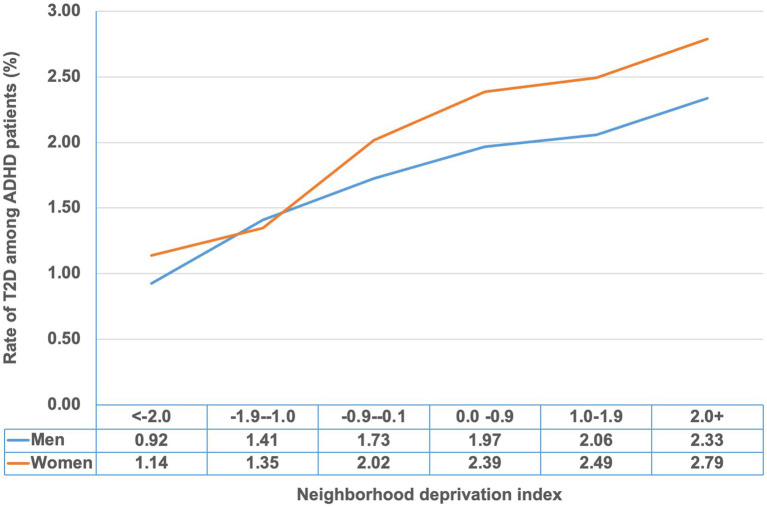
Cumulative rate of type 2 diabetes (%) among ADHD patients by neighborhood deprivation index.

Figure 1 shows the cumulative prevalence of T2D (%) among ADHD patients by sex and stratified by the neighborhood deprivation index. The prevalence increased with higher levels of neighborhood deprivation.

**Figure 2:**
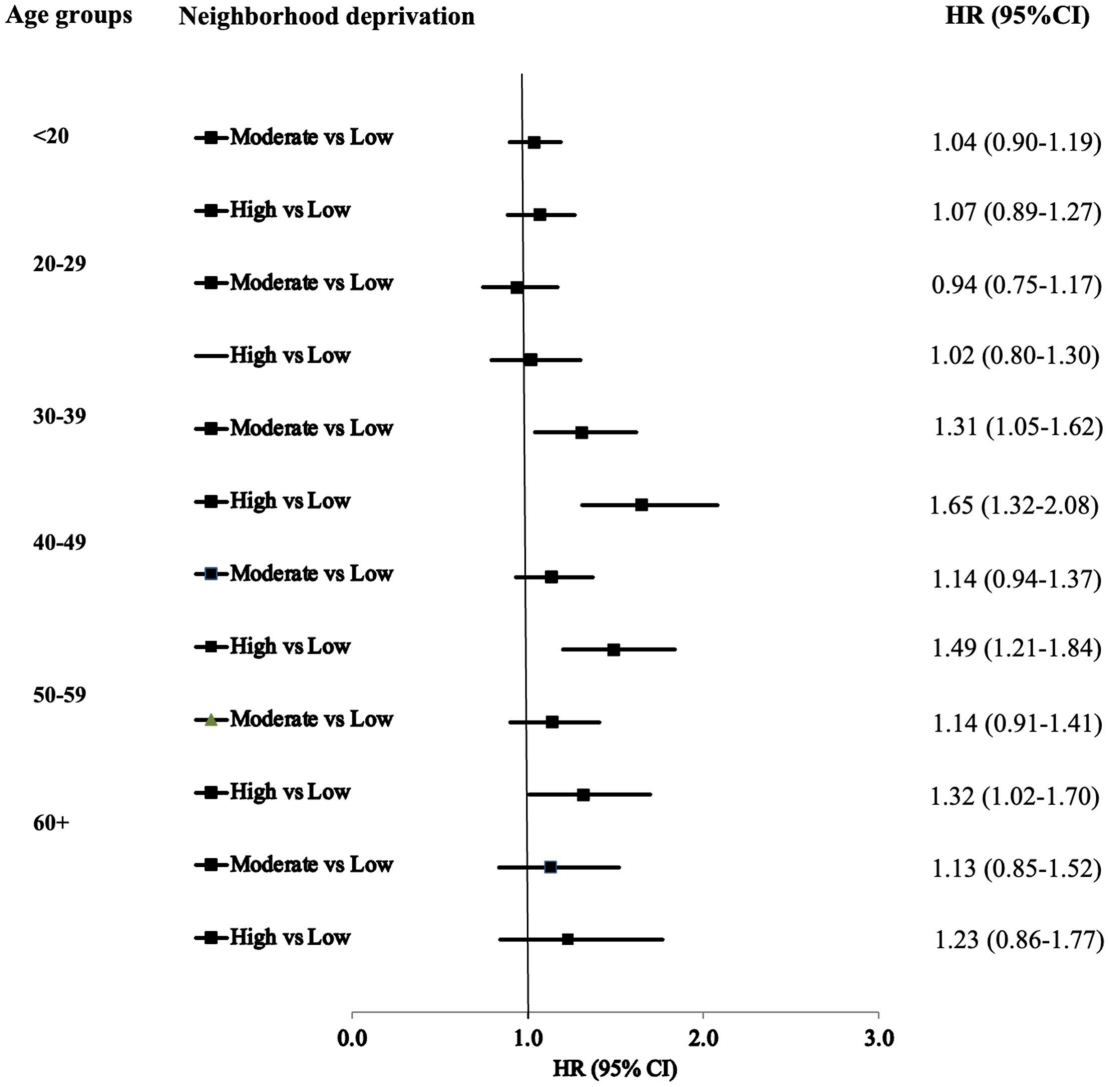
Hazards ratios (HR*) and 95% confidence intervals (CI) for type 2 diabetes of ADHD by age at diagnosis. *: Fully adjusted.

Figure 2 illustrates the HRs for T2D by age at ADHD diagnosis. The results suggest that the gradient of T2D diagnoses among ADHD patients became more pronounced with increasing neighborhood deprivation across different age groups. In the fully adjusted model for the 30–39 age group, the HRs were 1.31 (95% CI: 1.05–1.62) and 1.65 (95% CI: 1.32–2.08) in moderate- and high-deprivation neighborhoods, respectively. The HRs for T2D were significantly higher for ADHD patients aged 40–49 residing in high-deprivation neighborhoods compared to those in low-deprivation neighborhoods.

Table 2 details the HRs for T2D among men with ADHD, showing a graded association where the incidence of T2D increases with higher levels of neighborhood deprivation. The HRs for men were 1.09 (95% CI: 0.98–1.20) and 1.37 (95% CI: 1.22–1.53) in moderate- and high-deprivation neighborhoods, respectively. In the fully adjusted model, the associations were slightly attenuated but remained significant for high-deprivation neighborhoods, with a HR of 1.19 (95% CI: 1.06–1.34).

**Table 2:** Hazards ratios (HR) and 95% confidence intervals (CI) for type 2 diabetes in men; results of Cox regression models.

	Model 1			Model 2			Model 3			
	HR	95% CI		HR	95% CI		HR	95% CI		*p*-value
Neighborhood deprivation (ref. low)										
Moderate	1.09	0.98	1.20	1.02	0.92	1.13	0.98	0.88	1.09	0.7177
High	1.37	1.22	1.53	1.24	1.11	1.40	1.19	1.06	1.34	0.0033
Age	1.06	1.06	1.06	1.05	1.05	1.05	1.05	1.05	1.06	<0.0001
Family income (ref. highest quartiles)										
Low				0.94	0.83	1.06	0.93	0.83	1.05	0.2615
Middle-low				1.14	1.02	1.27	1.09	0.97	1.22	0.1451
Middle-high				1.02	0.91	1.15	0.98	0.87	1.10	0.7348
Education attainment in father (ref. ≥12 years)										
≤9 years				1.15	1.03	1.29	1.12	1.00	1.25	0.0572
10–11 years				1.05	0.93	1.18	1.03	0.91	1.16	0.6778
Education attainment in mother (ref. ≥12 years)										
≤9 years				1.09	0.97	1.22	1.07	0.95	1.19	0.2720
10–11 years				1.03	0.92	1.15	0.99	0.89	1.11	0.8655
Country of origin (ref. Sweden)				1.52	1.34	1.73	1.56	1.37	1.78	<0.0001
Country of origin in father (ref. Sweden)				0.89	0.78	1.01	0.88	0.77	1.01	0.0605
Country of origin in mother (ref. Sweden)				0.97	0.85	1.11	0.95	0.84	1.09	0.4596
Marital status in parents (ref. married/cohabiting)				0.93	0.86	1.01	0.92	0.85	1.00	0.0594
Region of residence (ref. large cities)										
Southern Sweden				1.12	1.03	1.22	1.13	1.03	1.23	0.0070
Northern Sweden				1.11	1.00	1.24	1.10	0.99	1.23	0.0909
Family history of type 2 diabetes (ref. non)				2.26	2.07	2.47	2.04	1.87	2.23	<0.0001
Hospitalization of obesity (ref. non)							5.09	4.61	5.62	<0.0001
Hospitalization of depression (ref. non)							1.23	1.13	1.34	<0.0001
Hospitalization of anxiety (ref. non)							1.25	1.15	1.36	<0.0001

HR, hazard ratio; CI, confidence interval. Model 1: Univariate model, adjusted for age. Model 2. Adjusted for individual characteristics. Model 3. Model 2 + comorbidities.

Table 3 shows the corresponding HRs for T2D in women with ADHD also indicating a graded association. The HRs associated with T2D were 1.44 (95% CI: 1.27–1.64) and 1.84 (95% CI: 1.61–2.12) for women living in moderate- and high-deprivation neighborhoods, respectively. Although the HRs decreased after adjusting for individual-level variables in the full model, they remained significant in both moderate-deprivation neighborhoods (HR = 1.30, 95% CI = 1.14–1.47) and high-deprivation neighborhoods (HR = 1.48, 95% CI = 1.28–1.70).

**Table 3:** Hazards ratios (HR) and 95% confidence intervals (CI) for type 2 diabetes in women; results of Cox regression.

	Model 1			Model 2			Model 3			
	HR	95% CI		HR	95% CI		HR	95% CI		*p*-value
Neighborhood deprivation (ref. low)										
Moderate	1.44	1.27	1.64	1.35	1.19	1.53	1.30	1.14	1.47	<0.0001
High	1.84	1.61	2.12	1.61	1.40	1.86	1.48	1.28	1.70	<0.0001
Age	1.04	1.04	1.04	1.03	1.03	1.04	1.04	1.03	1.04	<0.0001
Family income (ref. Highest quartiles)										
Low				1.18	1.01	1.37	1.11	0.95	1.30	0.1850
Middle-low				1.22	1.05	1.42	1.14	0.98	1.33	0.0816
Middle-high				1.08	0.92	1.26	1.04	0.89	1.21	0.6665
Education attainment in father (ref. ≥12 years)										
≤9 years				1.22	1.07	1.39	1.17	1.02	1.33	0.0213
10–11 years				1.17	1.02	1.35	1.10	0.96	1.27	0.1637
Education attainment in mother (ref. ≥12 years)										
≤9 years				1.14	1.00	1.30	1.10	0.97	1.25	0.1498
10–11 years				1.07	0.94	1.21	0.99	0.88	1.13	0.9239
Country of origin (ref. Sweden)				1.47	1.27	1.70	1.46	1.26	1.69	<0.0001
Country of origin in father (ref. Sweden)				0.97	0.84	1.12	0.97	0.84	1.13	0.7255
Country of origin in mother (ref. Sweden)				1.13	0.98	1.31	1.12	0.97	1.30	0.1217
Marital status in parents (ref. Married/cohabiting)				0.89	0.81	0.98	0.88	0.80	0.97	0.0106
Region of residence (ref. large cities)										
Southern Sweden				1.06	0.96	1.17	1.04	0.94	1.15	0.4757
Northern Sweden				1.05	0.93	1.19	1.06	0.94	1.21	0.3442
Family history of type 2 diabetes (ref. non)				2.17	1.96	2.40	1.88	1.69	2.08	<0.0001
Hospitalization of obesity (ref. non)							4.17	3.78	4.59	<0.0001
Hospitalization of depression (ref. non)							1.05	0.96	1.16	0.2954
Hospitalization of anxiety (ref. non)							1.25	1.13	1.38	<0.0001

HR, hazard ratio; CI, confidence interval. Model 1: Univariate model, adjusted for age. Model 2: Adjusted for individual characteristics. Model 3: Model 2 + comorbidities.

Supplementary Table S2 shows a clear gradient, with higher T2D incidence associated with increasing neighborhood deprivation in the total study population. The same pattern was observed across most subgroups. Additionally, the cumulative probability of not dying from T2D over the follow-up period was lower for patients residing in high-deprivation neighborhoods (Supplementary Figure S2).

Supplementary Table S3 includes all patients with ADHD and shows a similar association between neighborhood deprivation and incident T2D as seen in the sex-stratified analyses. For instance, the fully adjusted HRs for T2D were 1.11 (95% CI: 1.02–1.20) and 1.31 (95% CI: 1.20–1.43) for moderate- and high-deprivation neighborhoods compared to low-deprivation neighborhoods, respectively. Furthermore, individual-level variables were significantly associated with T2D in the fully adjusted models. For example, HRs for T2D were higher in men than women, in patients born outside Sweden, and in those with comorbidities and the risk of T2D increased with advancing age.

Supplementary Table S4 shows an additional fully adjusted sensitivity analysis, the study population of men and women with ADHD were identified from hospitalization and medication treatment, separately. Neighborhood deprivation was significantly associated with T2D.

In an additional fully adjusted sensitivity analysis (Supplementary Table S5)—in which a sub-sample of study population of men and women with ADHD were included, T2D were identified from hospitalization and medication treatment, separately, and followed from 2005 to 2018. Neighborhood deprivation was significantly associated with both diagnosis of T2D and medical treatment for T2D. For example, in this model, the fully adjusted HR for diagnosis of T2D with ADHD associated with high neighborhood deprivation was 1.41 (1.21 to 1.64) compared to low neighborhood deprivation. The corresponding HRs for medical treatment of T2D was 1.23 (1.09 to 1.39).

Additionally, we performed further analysis after excluding individuals who changed residential neighborhoods. Specifically, compared to low neighborhood deprivation, the fully adjusted HRs for T2D in highly deprived neighborhoods were 1.33 (95% CI = 1.20–1.47) (Supplementary Table S6). Another additional analysis showed that the association between neighborhood deprivation and T2D remained consistent regardless of the number of diagnoses for ADHD (Supplementary Table S7). Finally, we also found a statistically significant interaction for sex where neighborhood deprivation (p for interaction = 0.0229) was more strongly associated with T2D in women.

## Discussion

The main finding of this study was that neighborhood deprivation exhibited a graded association with incident T2D in both men and women with ADHD, with higher incidence rates of T2D observed as neighborhood deprivation increased. Although this association was attenuated after adjusting for individual-level sociodemographic variables and traditional risk factors for T2D (e.g., obesity), it remained statistically significant. The novel contribution of this study is that it provides evidence that the incidence rate of T2D increases with the level of neighborhood deprivation among patients with ADHD. These findings suggest that neighborhood deprivation may be considered an independent risk factor for T2D in both men and women with ADHD.

Systematic reviews have demonstrated that diabetes and related factors, including obesity, metabolic syndrome, and lifestyle behaviors, contribute significantly to the health disparities observed between individuals with ADHD and the general population (22–26). Several risk factors for T2D are more prevalent among individuals with ADHD than in the general population, which may partially explain their increased susceptibility to T2D. Furthermore, residing in highly deprived neighborhoods has been associated with a heightened risk of various morbidities, including ADHD (8) and T2D (15). Consistent with these findings, our study revealed that the incidence rates of T2D among individuals with ADHD increased in tandem with the level of neighborhood deprivation.

Social disparities in both the prevalence of T2D and impaired glucose regulation have been documented (27), and the association between neighborhood-level deprivation and T2D is well established (10). Previous studies have consistently shown that T2D prevalence is higher in deprived neighborhoods compared to affluent ones, even after adjusting for individual-level characteristics (10, 15, 28). Moreover, residents of highly deprived neighborhoods tend to experience a greater burden of T2D and cardiovascular disease risk factors, including physical inactivity, obesity, and tobacco use (29).

The causal pathways between neighborhood deprivation and ADHD are not fully understood (15, 30–32). However, multiple potential mechanisms may account for our findings. Variations in lifestyle attitudes and beliefs across socioeconomic status (SES) levels among patients with ADHD may significantly contribute to these findings (33–35). For example, a systematic reported a higher prevalence of smoking among patients with diabetes mellitus residing in deprived neighborhoods compared to those in more affluent areas (34). A similar pattern was observed in another neighborhood-based study examining T2D risk factors among patients with ADHD (35). Additionally, sociocultural norms related to diet, smoking, and physical activity may differ across neighborhoods, influencing residents’ health and their subsequent disease risk.

Moreover, a previous study conducted by our group in Sweden found that the availability of potentially health-promoting goods, services, and resources is, in fact, higher in more deprived neighborhoods compared to affluent ones. However, the same trend applies to health-damaging neighborhood features, which may partially explain the adverse health outcomes observed in deprived areas (16). On the other hand, while Sweden’s healthcare system ensures geographical access even in deprived areas, actual healthcare utilization remains an individual-level behavior that may be influenced by socioeconomic, cultural, and psychological factors—such as distrust of medical institutions, limited health literacy, or comorbid mental illness. It is also possible that interactions between the two levels may occur, i.e., community-level deprivation may interact with individual behaviors to shape T2D risk, despite nominally equal access to healthcare (16). These disparities may stem from individual sociodemographic factors influencing patients’ ability to afford prescribed medications (36) and from limited actual access to primary healthcare in deprived neighborhoods (37), which could, in turn, hinder preventive care for T2D. To examine this further, we conducted a sensitivity analysis using both diagnostic records and prescriptions for T2D medication. The persistence of findings across both outcome definitions suggests a high reliability of our results.

An additional finding of this study was that women with ADHD appeared to be more affected by neighborhood deprivation than men with ADHD concerning the incidence of T2D. Generally, women may spend more time in their immediate neighborhoods than men. Several factors could explain these findings. For instance, differences in comorbidity distribution and healthcare-seeking behaviors may exist between men and women. Further research is needed to explain these findings more closely.

Furthermore, divergent associations with incident T2D were observed for certain individual-level sociodemographic factors (e.g., family income) and comorbidities (e.g., anxiety, depression, and obesity), which may, among other factors, be attributable to variations in healthcare-seeking behaviors. For example, individuals with ADHD and low income and/or certain comorbidities may be more or less likely to seek healthcare for T2D symptoms, potentially influencing the incidence rates of T2D. However, further research is needed to validate our findings in different settings and to explore the underlying mechanisms driving the observed discrepancies in T2D incidence.

The present study has several important limitations. Most notably, we lacked data on key risk factors for T2D, including smoking, high-calorie diet, and physical inactivity. However, we attempted to mitigate this limitation by adjusting for comorbidities that could serve as proxies for these potential confounders (e.g., obesity as a proxy for a high-calorie diet and physical inactivity). The findings remained significant but were slightly attenuated after these adjustments. Moreover, previous studies on socioeconomic status and T2D that accounted for smoking and physical inactivity have still identified an independent association.

Secondly, although Sweden has a universal healthcare system, ensuring that most patients with ADHD receive a diagnosis, some cases may have been overlooked if individuals did not seek medical care. Moreover, it is not possible to rule out that a diagnosis of ADHD could have led to a surveillance bias if ADHD patients would be more likely to be investigated for T2D than those without the condition. Another potential limitation of studies like ours is that a substantial number of patients may have changed residence and neighborhood deprivation status during the study period. Residential mobility (i.e., relocation between neighborhoods with different levels of deprivation) may influence findings in studies examining neighborhood deprivation and health. However, we did not adjust for mobility, as only a relatively limited number of incidents T2D cases (i.e., 17.3% events) were observed among individuals who relocated during the study period. To remedy this, we also examined the association between neighborhood deprivation and T2D after excluding those ADHD patients who relocated and found that the associations remained consistent. Finally, we lacked data on neighborhood healthcare quality, preventing us from evaluating whether this factor played a significant role in our findings.

Nevertheless, these limitations are offset by several notable strengths. First, the cohort was substantial, encompassing nearly all patients with ADHD in Sweden during the study period, thereby enhancing the generalizability of our findings. Another strength was the use of personal identification numbers (pseudonymized in this study), which are assigned to all individuals in Sweden and allowed us to track participants with minimal loss to follow-up. Third, the outcome data were derived from clinical diagnoses recorded by physicians rather than self-reported data, thereby eliminating recall bias.

An additional key strength was access to data from SAMS units. These units delineated small geographic boundaries of neighborhoods with relatively homogeneous building types, each encompassing approximately 1,000–2,000 residents. The small size of these units was an advantage, as previous research has shown that small neighborhoods align well with residents’ own perceptions of their communities. Moreover, our dataset was highly complete, with only 185 study participants excluded due to missing SAMS codes. National demographic and individual sociodemographic data were also nearly complete, with less than 0.1% missing. This allowed us to use linked clinical data from individual patients with comprehensive national demographic and socioeconomic records.

The present findings, along with previous evidence, highlights the need to improve health and healthcare in low resource settings, which have been underway in Europe (38). However, when addressing the well-known health disparities in the deprived and disadvantaged (39), an evidence based approach is vital. Studies like the present study, which identifies specific groups of patients in deprived neighborhoods that are in greatest need of additional healthcare recourses, are important for targeted interventions. We also suggest that clinicians use our findings when treating patients with ADHD.

## Conclusion

The findings of this study are valuable for healthcare professionals working with patients with ADHD, particularly those residing in deprived neighborhoods. Understanding the pathways linking neighborhood factors—independent of individual characteristics—to various health outcomes remains a challenge. Future research should investigate the specific pathways between neighborhood environments and T2D, as well as strategies to mitigate disparities in T2D among patients with ADHD across different neighborhood settings. Such research is essential for identifying mechanisms that could inform effective preventive strategies and health policies.

This study identified patients with ADHD living in deprived neighborhoods as a particularly vulnerable group for developing T2D. By integrating individual and contextual risk factors, our findings can help policymakers target diabetes prevention strategies more precisely, e.g., tailored health education, screening initiatives, and community-based interventions in high-deprivation areas. Our study also underscores the need to align psychiatric care with metabolic disease prevention, especially in socioeconomically deprived contexts.

## Data Availability

The original contributions presented in the study are included in the article/Supplementary material, further inquiries can be directed to the corresponding author.
