# Supplementary material for: Association between neighborhood deprivation and type 2 diabetes risk among ADHD patients: a nationwide population-based cohort study
Source: Front Public Health. 2025 Sep 11;13:1609551. doi: 10.3389/fpubh.2025.1609551 (PMC12460135; doi:10.3389/fpubh.2025.1609551)

**Supplementary materials:**

**Table S1. Study population and neighborhoods**

**Table S2. Distribution of population. number of cases. and cumulative rates (per 100) of type 2 diabetes of ADHD patients. 2001-2018**

**Table S3. Hazards ratios (HR) and 95% confidence intervals (CI) for type 2 diabetes; Results of Cox regression models**

**Table S4. Hazards ratios (HR) and 95% confidence intervals (CI) for type 2 diabetes; Results of Cox regression models, patients were identified in hospitalizations and medication treatments**

**Table S5. Hazards ratios (HR) and 95% confidence intervals (CI) for diagnosis and treatment of type 2 diabetes; Results of Cox regression models**

**Table S6. Hazards ratios (HR) and 95% confidence intervals (CI) for type 2 diabetes, after excluding individuals who moved residences during the study period; Results of Cox regression models**

**Table S7. Hazards ratios (HR) and 95% confidence intervals (CI) for type 2 diabetes by grade of ADHD; Results of Cox regression models**

**Figure S1. Flow-chart of study population**

**Figure S2. Kaplan-Meier survival estimates of T2D among ADHD patients.**

| **Table S1. Study population and neighborhoods** | |  |  |  |
| --- | --- | --- | --- | --- |
|  | Low | Moderate | High | Total |
| Number of neighborhoods (%) | 1709 (27.7) | 3285 (53.1) | 1189 (19.2) | 6183 |
| SES index | <-1 | -1-1 | >1 |  |
| Study population (%) | 63000 (25.5) | 129354 (52.5) | 54161 (22.0) | 246515 |
| Events of type 2 diabetes (%) | 836 (18.3) | 2482 (52.2) | 1257 (27.5) | 4575 |

| **Table S2. Distribution of population. number of cases. and cumulative rates (per 100) of type 2 diabetes of ADHD patients. 2001-2018** | | | | | | | | | |
| --- | --- | --- | --- | --- | --- | --- | --- | --- | --- |
|  | Population | |  | Type 2 diabetes cases | |  | Cumulative rates (%) of diabetes by neighborhood deprivation | | |
|  | No. | % |  | No. | % |  | Low (n=63000) | Moderate (n=129354) | High (n=54161) |
| Total population | 246515 |  |  | 4575 |  |  | 1.3 | 1.9 | 2.3 |
| **Gender** |  |  |  |  |  |  |  |  |  |
| Males | 149242 | 60.5 |  | 2622 | 57.3 |  | 1.4 | 1.8 | 2.2 |
| Females | 97273 | 39.5 |  | 1953 | 42.7 |  | 1.3 | 2.1 | 2.6 |
| **Age (years)** |  |  |  |  |  |  |  |  |  |
| <20 | 139949 | 56.8 |  | 1293 | 28.3 |  | 0.8 | 1.0 | 1.0 |
| 20-29 | 43576 | 17.7 |  | 651 | 14.2 |  | 1.1 | 1.5 | 1.8 |
| 30-39 | 29976 | 12.2 |  | 829 | 18.1 |  | 1.7 | 2.8 | 3.7 |
| 40-49 | 20674 | 8.4 |  | 917 | 20.0 |  | 2.9 | 4.5 | 5.9 |
| 50-59 | 9118 | 3.7 |  | 592 | 12.9 |  | 4.9 | 6.7 | 7.8 |
| ≥ 60 | 3222 | 1.3 |  | 293 | 6.4 |  | 8.0 | 9.6 | 9.1 |
| **Paternal educational level (years)** |  |  |  |  |  |  |  |  |  |
| ≤ 9 | 88718 | 36.0 |  | 2583 | 56.5 |  | 2.4 | 3.0 | 3.2 |
| 10-11 | 75156 | 30.5 |  | 1086 | 23.7 |  | 1.0 | 1.5 | 1.8 |
| ≥ 12 | 82641 | 33.5 |  | 906 | 19.8 |  | 0.9 | 1.2 | 1.4 |
| **Maternal educational level (years)** |  |  |  |  |  |  |  |  |  |
| ≤ 9 | 64921 | 26.3 |  | 2105 | 46.0 |  | 2.8 | 3.4 | 3.3 |
| 10-11 | 70238 | 28.5 |  | 1291 | 28.2 |  | 1.2 | 1.9 | 2.2 |
| ≥ 12 | 111356 | 45.2 |  | 1179 | 25.8 |  | 0.9 | 1.1 | 1.4 |
| **Family income** |  |  |  |  |  |  |  |  |  |
| Low | 61507 | 25.0 |  | 1197 | 26.2 |  | 1.5 | 2.0 | 2.1 |
| Middle-low | 61669 | 25.0 |  | 1521 | 33.2 |  | 1.8 | 2.4 | 3.1 |
| Middle-high | 61706 | 25.0 |  | 1077 | 23.5 |  | 1.3 | 1.8 | 2.2 |
| High | 61633 | 25.0 |  | 780 | 17.0 |  | 1.0 | 1.4 | 1.5 |
| **Region of residence** |  |  |  |  |  |  |  |  |  |
| Large cities | 132921 | 53.9 |  | 2270 | 49.6 |  | 1.3 | 1.8 | 2.1 |
| Southern Sweden | 76625 | 31.1 |  | 1544 | 33.7 |  | 1.4 | 2.0 | 2.5 |
| Northern Sweden | 36969 | 15.0 |  | 761 | 16.6 |  | 1.5 | 2.0 | 2.8 |
| **Marital status in parent** |  |  |  |  |  |  |  |  |  |
| Married/cohabiting | 127739 | 51.8 |  | 2689 | 58.8 |  | 1.5 | 2.2 | 2.8 |
| Not married | 118776 | 48.2 |  | 1886 | 41.2 |  | 1.1 | 1.6 | 1.9 |
| **Immigrant status** |  |  |  |  |  |  |  |  |  |
| Born in Sweden | 226137 | 91.7 |  | 3979 | 87.0 |  | 1.3 | 1.8 | 2.2 |
| Born in other countries | 20378 | 8.3 |  | 596 | 13.0 |  | 2.3 | 3.0 | 3.2 |
| **Immigrant status in father** |  |  |  |  |  |  |  |  |  |
| Born in Sweden | 205880 | 83.5 |  | 3913 | 85.5 |  | 1.3 | 2.0 | 2.5 |
| Born in other countries | 40635 | 16.5 |  | 662 | 14.5 |  | 1.4 | 1.6 | 1.8 |
| **Immigrant status in mother** |  |  |  |  |  |  |  |  |  |
| Born in Sweden | 208713 | 84.7 |  | 3889 | 85.0 |  | 1.3 | 1.9 | 2.5 |
| Born in other countries | 37802 | 15.3 |  | 686 | 15.0 |  | 1.4 | 1.9 | 1.9 |
| **Family history of type 2 diabetes** |  |  |  |  |  |  |  |  |  |
| Non | 222009 | 90.1 |  | 3342 | 73.0 |  | 1.1 | 1.6 | 1.8 |
| Yes | 24506 | 9.9 |  | 1233 | 27.0 |  | 3.9 | 5.1 | 5.7 |
| **Hospitalization of obesity** |  |  |  |  |  |  |  |  |  |
| Non | 231293 | 93.8 |  | 3451 | 75.4 |  | 1.1 | 1.5 | 1.8 |
| Yes | 15222 | 6.2 |  | 1124 | 24.6 |  | 5.9 | 7.5 | 8.2 |
| **Hospitalization of depression** |  |  |  |  |  |  |  |  |  |
| Non | 178436 | 72.4 |  | 2582 | 56.4 |  | 1.1 | 1.5 | 1.8 |
| Yes | 68079 | 27.6 |  | 1993 | 43.6 |  | 2.1 | 3.0 | 3.7 |
| **Hospitalization of anxiety** |  |  |  |  |  |  |  |  |  |
| Non | 155063 | 62.9 |  | 2091 | 45.7 |  | 1.0 | 1.4 | 1.6 |
| Yes | 91452 | 37.1 |  | 2484 | 54.3 |  | 2.0 | 2.7 | 3.4 |

| **Table S3. Hazards ratios (HR) and 95% confidence intervals (CI) for type 2 diabetes; Results of Cox regression models** | | | | | | | | | |
| --- | --- | --- | --- | --- | --- | --- | --- | --- | --- |
|  | Crude model | | | |  | Fully adjusted | | | |
|  | HR^a^ | 95% CI | | P-value |  | HR^b^ | 95% CI | | P-value |
| Neighbourhood deprivation (ref. Low) |  |  |  |  |  |  |  |  |  |
| Moderate | 1.23 | 1.13 | 1.33 | <.0001 |  | 1.11 | 1.02 | 1.20 | 0.0140 |
| High | 1.56 | 1.43 | 1.70 | <.0001 |  | 1.31 | 1.20 | 1.43 | <.0001 |
| Age | 1.05 | 1.05 | 1.05 | <.0001 |  | 1.05 | 1.04 | 1.05 | <.0001 |
| Gender to males (ref. Females) | 0.98 | 0.93 | 1.04 | 0.5801 |  | 1.22 | 1.15 | 1.30 | <.0001 |
| Family income (ref. Highest quartiles) |  |  |  |  |  |  |  |  |  |
| Low | 1.20 | 1.09 | 1.31 | <.0001 |  | 0.99 | 0.90 | 1.09 | 0.8527 |
| Middle-low | 1.30 | 1.19 | 1.41 | <.0001 |  | 1.08 | 0.99 | 1.18 | 0.0831 |
| Middle-high | 1.10 | 1.00 | 1.21 | 0.0459 |  | 0.98 | 0.89 | 1.07 | 0.6126 |
| Education attainment in father (ref. ≥ 12 years) |  |  |  |  |  |  |  |  |  |
| ≤ 9 years | 1.31 | 1.21 | 1.42 | <.0001 |  | 1.14 | 1.04 | 1.24 | 0.0033 |
| 10–11 years | 1.16 | 1.06 | 1.27 | <.0001 |  | 1.06 | 0.97 | 1.16 | 0.2227 |
| Education attainment in mother (ref. ≥ 12 years) |  |  |  | <.0001 |  |  |  |  |  |
| ≤ 9 years | 1.35 | 1.25 | 1.46 | <.0001 |  | 1.08 | 0.99 | 1.17 | 0.0844 |
| 10–11 years | 1.17 | 1.08 | 1.27 | <.0001 |  | 0.99 | 0.91 | 1.08 | 0.8375 |
| Country of origin (ref. Sweden) | 1.46 | 1.34 | 1.59 | <.0001 |  | 1.51 | 1.37 | 1.66 | <.0001 |
| Country of origin in father (ref. Sweden) | 1.09 | 1.00 | 1.18 | 0.0462 |  | 0.92 | 0.84 | 1.01 | 0.0897 |
| Country of origin in mother (ref. Sweden) | 1.17 | 1.08 | 1.27 | 0.0001 |  | 1.02 | 0.92 | 1.12 | 0.7531 |
| Marital status in parents (ref. Married/cohabiting) | 0.93 | 0.88 | 0.99 | 0.0186 |  | 0.91 | 0.85 | 0.97 | 0.0021 |
| Region of residence (ref. Large cities) |  |  |  |  |  |  |  |  |  |
| Southern Sweden | 1.13 | 1.06 | 1.20 | 0.0003 |  | 1.09 | 1.02 | 1.16 | 0.0108 |
| Northern Sweden | 1.09 | 1.00 | 1.18 | 0.0447 |  | 1.08 | 1.00 | 1.18 | 0.0614 |
| Family history of type 2 diabetes (ref. Non) | 2.18 | 2.04 | 2.33 | <.0001 |  | 1.98 | 1.85 | 2.12 | <.0001 |
| Hospitalization of obesity (ref. Non) | 4.93 | 4.61 | 5.28 | <.0001 |  | 4.52 | 4.21 | 4.84 | <.0001 |
| Hospitalization of depression (ref. Non) | 1.38 | 1.30 | 1.46 | <.0001 |  | 1.16 | 1.09 | 1.24 | <.0001 |
| Hospitalization of anxiety (ref. Non) | 1.46 | 1.38 | 1.55 | <.0001 |  | 1.28 | 1.20 | 1.36 | <.0001 |
| HR: Hazard ratio; CI: Confidence interval.  a: Crude model: adjusted for age; b: Fully adjusted. |  |  |  |  |  |  |  |  |  |

| **Table S4. Hazards ratios (HR) and 95% confidence intervals (CI) for diabetes; Results of Cox regression models** | | | | | | | | | |
| --- | --- | --- | --- | --- | --- | --- | --- | --- | --- |
|  | Hospitalization of ADHD | | | |  | Medication of ADHD | | | |
|  | HR* | 95% CI | | P-value |  | HR* | 95% CI | | P-value |
| Neighborhood deprivation (ref. Low) |  |  |  |  |  |  |  |  |  |
| Moderate | 1.13 | 1.03 | 1.24 | 0.0123 |  | 1.04 | 0.90 | 1.21 | 0.5694 |
| High | 1.30 | 1.17 | 1.45 | <.0001 |  | 1.34 | 1.13 | 1.60 | 0.0009 |
| Gender to males (ref. Females) | 1.25 | 1.16 | 1.34 | <.0001 |  | 1.15 | 1.02 | 1.30 | 0.0245 |
| Age | 1.05 | 1.04 | 1.05 | <.0001 |  | 1.04 | 1.04 | 1.04 | <.0001 |
| Family income (ref. Highest quartiles) |  |  |  |  |  |  |  |  |  |
| Low | 1.05 | 0.94 | 1.18 | 0.3770 |  | 0.84 | 0.70 | 1.01 | 0.0627 |
| Middle-low | 1.11 | 1.00 | 1.24 | 0.0530 |  | 1.02 | 0.87 | 1.21 | 0.7850 |
| Middle-high | 1.03 | 0.92 | 1.15 | 0.6375 |  | 0.86 | 0.72 | 1.03 | 0.0918 |
| Education attainment in father (ref. ≥ 12 years) |  |  |  |  |  |  |  |  |  |
| ≤ 9 years | 1.11 | 1.01 | 1.23 | 0.0319 |  | 1.20 | 1.01 | 1.43 | 0.0388 |
| 10–11 years | 1.07 | 0.96 | 1.18 | 0.2293 |  | 1.02 | 0.85 | 1.24 | 0.8097 |
| Education attainment in mother (ref. ≥ 12 years) |  |  |  |  |  |  |  |  |  |
| ≤ 9 years | 1.04 | 0.94 | 1.14 | 0.4765 |  | 1.22 | 1.03 | 1.45 | 0.0231 |
| 10–11 years | 0.99 | 0.90 | 1.09 | 0.8261 |  | 0.98 | 0.83 | 1.16 | 0.8357 |
| Country of origin (ref. Sweden) | 1.47 | 1.31 | 1.64 | <.0001 |  | 1.60 | 1.33 | 1.93 | <.0001 |
| Country of origin in father (ref. Sweden) | 1.00 | 0.89 | 1.11 | 0.9470 |  | 0.70 | 0.57 | 0.86 | 0.0009 |
| Country of origin in mother (ref. Sweden) | 0.97 | 0.87 | 1.09 | 0.6107 |  | 1.17 | 0.96 | 1.41 | 0.1163 |
| Marital status in parents (ref. Married/cohabiting) | 0.90 | 0.84 | 0.97 | 0.0053 |  | 0.92 | 0.81 | 1.05 | 0.2009 |
| Region of residence (ref. Large cities) |  |  |  |  |  |  |  |  |  |
| Southern Sweden | 1.09 | 1.01 | 1.18 | 0.0197 |  | 1.06 | 0.92 | 1.22 | 0.4317 |
| Northern Sweden | 1.08 | 0.98 | 1.19 | 0.1469 |  | 1.11 | 0.94 | 1.30 | 0.2125 |
| Family history of type 2 diabetes (ref. Non) | 2.01 | 1.86 | 2.17 | <.0001 |  | 1.88 | 1.64 | 2.16 | <.0001 |
| Hospitalization of obesity (ref. Non) | 4.44 | 4.10 | 4.81 | <.0001 |  | 4.80 | 4.18 | 5.51 | <.0001 |
| Hospitalization of depression (ref. Non) | 1.16 | 1.07 | 1.25 | 0.0001 |  | 1.16 | 1.02 | 1.33 | 0.0253 |
| Hospitalization of anxiety (ref. Non) | 1.34 | 1.24 | 1.44 | <.0001 |  | 1.10 | 0.97 | 1.26 | 0.1363 |
| HR: Hazard ratio; CI: Confidence interval. |  |  |  |  |  |  |  |  |  |
| *: Fully adjusted. |  |  |  |  |  |  |  |  |  |

| **Table S5. Hazards ratios (HR) and 95% confidence intervals (CI) for diagnosis and treatment of diabetes; Results of Cox regression models** | | | | | | | | | | |
| --- | --- | --- | --- | --- | --- | --- | --- | --- | --- | --- |
|  | Diagnosis of T2D. 2005-2018 | | | |  | Medication of T2D. 2005-2018 | | | |  |
|  | HR* | 95% CI | | P-value |  | HR* | 95% CI | | P-value |  |
| Neighborhood deprivation (ref. Low) |  |  |  |  |  |  |  |  |  |  |
| Moderate | 1.11 | 0.96 | 1.27 | 0.1529 |  | 1.11 | 1.00 | 1.23 | 0.0595 |  |
| High | 1.41 | 1.21 | 1.64 | <.0001 |  | 1.23 | 1.09 | 1.39 | 0.0007 |  |
| Gender to males (ref. Females) | 1.70 | 1.53 | 1.89 | <.0001 |  | 1.02 | 0.94 | 1.10 | 0.6695 |  |
| Age | 1.07 | 1.06 | 1.07 | <.0001 |  | 1.03 | 1.03 | 1.04 | <.0001 |  |
| Family income ( ref. Highest quartiles) |  |  |  |  |  |  |  |  |  |  |
| Low | 1.36 | 1.16 | 1.60 | 0.0002 |  | 0.87 | 0.77 | 0.98 | 0.0233 |  |
| Middle-low | 1.38 | 1.18 | 1.60 | <.0001 |  | 0.95 | 0.85 | 1.07 | 0.4007 |  |
| Middle-high | 1.08 | 0.92 | 1.27 | 0.3415 |  | 0.94 | 0.84 | 1.06 | 0.3298 |  |
| Education attainment in father (ref. ≥ 12 years) |  |  |  |  |  |  |  |  |  |  |
| ≤ 9 years | 1.23 | 1.05 | 1.44 | 0.0095 |  | 1.11 | 0.99 | 1.24 | 0.0652 |  |
| 10–11 years | 1.20 | 1.01 | 1.42 | 0.0353 |  | 1.01 | 0.90 | 1.13 | 0.8800 |  |
| Education attainment in mother (ref. ≥ 12 years) |  |  |  |  |  |  |  |  |  |  |
| ≤ 9 years | 1.26 | 1.08 | 1.47 | 0.0027 |  | 1.02 | 0.91 | 1.14 | 0.7643 |  |
| 10–11 years | 1.08 | 0.92 | 1.25 | 0.3559 |  | 0.99 | 0.89 | 1.10 | 0.8541 |  |
| Country of origin (ref. Sweden) | 1.52 | 1.30 | 1.78 | <.0001 |  | 1.47 | 1.29 | 1.68 | <.0001 |  |
| Country of origin in father (ref. Sweden) | 0.86 | 0.73 | 1.02 | 0.0840 |  | 0.95 | 0.84 | 1.08 | 0.4193 |  |
| Country of origin in mother (ref. Sweden) | 1.08 | 0.92 | 1.27 | 0.3646 |  | 0.99 | 0.87 | 1.13 | 0.9022 |  |
| Marital status in parents (ref. Married/cohabiting) | 0.94 | 0.84 | 1.04 | 0.2281 |  | 0.93 | 0.85 | 1.00 | 0.0622 |  |
| Region of residence (ref. Large cities) |  |  |  |  |  |  |  |  |  |  |
| Southern Sweden | 1.10 | 0.99 | 1.23 | 0.0817 |  | 1.07 | 0.98 | 1.16 | 0.1475 |  |
| Northern Sweden | 1.13 | 0.99 | 1.30 | 0.0685 |  | 1.05 | 0.94 | 1.17 | 0.3979 |  |
| Family history of type 2 diabetes (ref. Non) | 2.26 | 2.03 | 2.52 | <.0001 |  | 1.81 | 1.65 | 1.98 | <.0001 |  |
| Hospitalization of obesity (ref. Non) | 5.29 | 4.72 | 5.93 | <.0001 |  | 4.17 | 3.80 | 4.58 | <.0001 |  |
| Hospitalization of depression (ref. Non) | 1.33 | 1.20 | 1.48 | <.0001 |  | 1.11 | 1.02 | 1.21 | 0.0179 |  |
| Hospitalization of anxiety (ref. Non) | 1.31 | 1.17 | 1.45 | <.0001 |  | 1.26 | 1.16 | 1.38 | <.0001 |  |
| HR: Hazard ratio; CI: Confidence interval. |  |  |  |  |  |  |  |  |  |  |
| *: Fully adjusted. |  |  |  |  |  |  |  |  |  |  |

| **Table S6. Hazards ratios (HR) and 95% confidence intervals (CI) for diabetes, after excluding individuals who moved residences during the study period; Results of Cox regression models.** | | | | |
| --- | --- | --- | --- | --- |
|  | HR* | 95% CI | | P-value |
| Neighborhood deprivation (ref. Low) |  |  |  |  |
| Moderate | 1.09 | 1.00 | 1.20 | 0.0509 |
| High | 1.33 | 1.20 | 1.47 | <.0001 |
| Age | 1.05 | 1.04 | 1.05 | <.0001 |
| Gender to males (ref. Females) | 1.22 | 1.14 | 1.30 | <.0001 |
| Family income ( ref. Highest quartiles) |  |  |  |  |
| Low | 1.02 | 0.92 | 1.13 | 0.6920 |
| Middle-low | 1.09 | 0.99 | 1.20 | 0.0734 |
| Middle-high | 1.00 | 0.90 | 1.10 | 0.9628 |
| Education attainment in father (ref. ≥ 12 years) |  |  |  |  |
| ≤ 9 years | 1.08 | 0.99 | 1.19 | 0.0982 |
| 10–11 years | 1.02 | 0.92 | 1.13 | 0.7072 |
| Education attainment in mother (ref. ≥ 12 years) |  |  |  |  |
| ≤ 9 years | 1.08 | 0.98 | 1.18 | 0.1309 |
| 10–11 years | 0.99 | 0.91 | 1.09 | 0.8928 |
| Country of origin (ref. Sweden) | 1.52 | 1.37 | 1.69 | <.0001 |
| Country of origin in father (ref. Sweden) | 0.93 | 0.83 | 1.03 | 0.1665 |
| Country of origin in mother (ref. Sweden) | 1.01 | 0.91 | 1.13 | 0.7987 |
| Marital status in parents (ref. Married/cohabiting) | 0.92 | 0.86 | 0.99 | 0.0233 |
| Region of residence (ref. Large cities) |  |  |  |  |
| Southern Sweden | 1.09 | 1.02 | 1.18 | 0.0160 |
| Northern Sweden | 1.09 | 0.99 | 1.19 | 0.0770 |
| Family history of type 2 diabetes (ref. Non) | 1.95 | 1.81 | 2.10 | <.0001 |
| Hospitalization of obesity (ref. Non) | 4.55 | 4.22 | 4.92 | <.0001 |
| Hospitalization of depression (ref. Non) | 1.12 | 1.05 | 1.21 | 0.0014 |
| Hospitalization of anxiety (ref. Non) | 1.29 | 1.20 | 1.38 | <.0001 |
| HR: Hazard ratio; CI: Confidence interval. |  |  |  |  |
| *: Fully adjusted. |  |  |  |  |

| **Table S7. Hazards ratios (HR) and 95% confidence intervals (CI) for diabetes by grade of ADHD; Results of Cox regression models** | | | | |
| --- | --- | --- | --- | --- |
|  | HR* | 95% CI | | P-value |
| **Number of diagnosis of ADHD=1 (N=58410)** |  |  |  |  |
| Neighborhood deprivation (ref. Low) |  |  |  |  |
| Moderate | 0.97 | 0.82 | 1.15 | 0.7212 |
| High | 1.32 | 1.09 | 1.60 | 0.0050 |
| **Number of diagnosis of ADHD 2-5 (N=70960)** |  |  |  |  |
| Neighborhood deprivation (ref. Low) |  |  |  |  |
| Moderate | 1.18 | 0.96 | 1.44 | 0.1133 |
| High | 1.57 | 1.26 | 1.96 | <.0001 |
| **Number of diagnosis of ADHD 6-12 (N=56834)** |  |  |  |  |
| Neighborhood deprivation (ref. Low) |  |  |  |  |
| Moderate | 1.20 | 0.99 | 1.45 | 0.1133 |
| High | 1.33 | 1.07 | 1.64 | <.0001 |
| **Number of diagnosis of ADHD over 12 (N=60311)** |  |  |  |  |
| Neighborhood deprivation (ref. Low) |  |  |  |  |
| Moderate | 1.14 | 1.01 | 1.29 | 0.0362 |
| High | 1.23 | 1.07 | 1.42 | 0.0046 |
| HR: Hazard ratio; CI: Confidence interval. |  |  |  |  |
| *: Fully adjusted. |  |  |  |  |

**Figure S1. Flow-chart of study population**

| Study population |  |
| --- | --- |
| 250386 | Excluding patients with unknown neighborhood deprivation index=185 |
| 250201 | Excluding previous diagnosis of diabetes. 1998-2000 =516 |
| 249685 | Excluding diabetes diagnosed before ADHD=3170 |
| 246515 | Final study population |

**Figure S2. Kaplan-Meier survival estimates of T2D among ADHD patients**


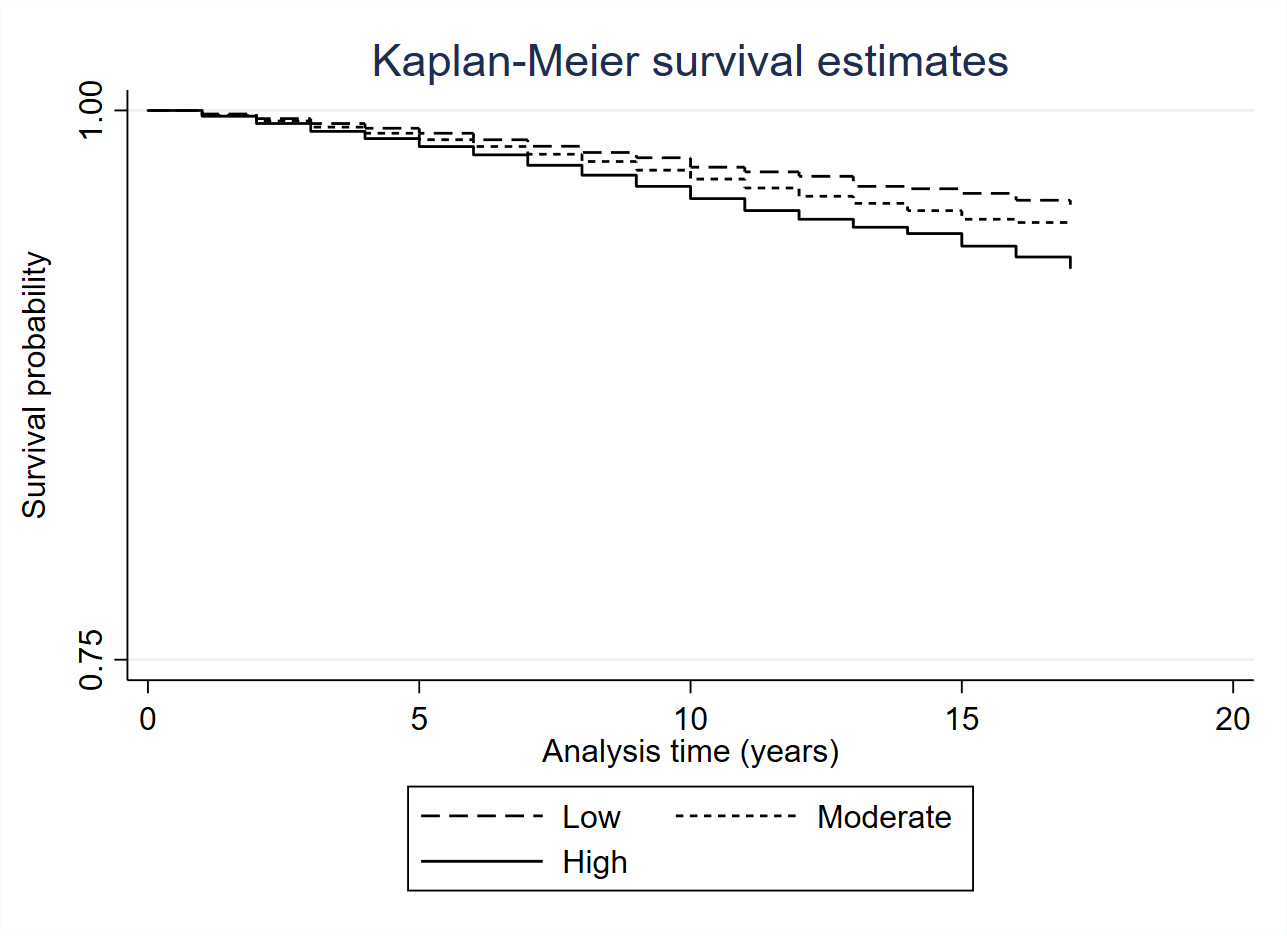

Supplement: Supplementary file 1 [file Table_1.DOCX]
